# Homicide pattern among adolescents: A national epidemiological study of child homicide in South Africa

**DOI:** 10.1371/journal.pone.0221415

**Published:** 2019-08-29

**Authors:** Shanaaz Mathews, Naeemah Abrahams, Lorna J. Martin, Carl Lombard, Rachel Jewkes

**Affiliations:** 1 Children’s Institute, Faculty of Health Sciences, University of Cape Town, Cape Town, South Africa; 2 Gender & Health Research Unit, South African Medical Research Council, Cape Town, South Africa; 3 The Division of Social and Behavioural Sciences: School of Public Health and Family Medicine, University of Cape Town, Cape Town, South Africa; 4 Forensic Pathology Services, Western Cape and Forensic Medicine & Toxicology, University of Cape Town, Cape Town, South Africa; 5 Biostatics Unit, South African Medical Research Council, Cape Town, South Africa; Stellenbosch University, SOUTH AFRICA

## Abstract

**Background:**

Large numbers of young people die yearly due to homicide, but little is known about homicide during adolescence. Research primarily focuses on youth violence among young men and masks important gender-related factors inherent in the adolescent age group. Although young women are less likely to be victims of homicide, violence against women is an important form of violence experienced during adolescence. In this paper, we describe the prevalence of and gender difference in adolescent homicide in South Africa in 2009.

**Methods:**

We conducted a retrospective national mortuary-based study to identify all child homicides (boys and girls < 18 years) in 2009 in a proportionate sample of mortuaries. Victim, perpetrator and crime data were collected in three phases: cases were identified from the mortuary register, the autopsy report and from police interviews. In this paper we focus on the adolescent group, aged 10 to 17 years.

**Findings:**

We identified 674 (95% CI: 520–823) adolescent homicides for 2009, with more male (520) than female (154) homicides. This gender disparity increased as children aged, with the male homicide rate 27.9/100 000 population (95% CI: 20.3–35.5), nearly 5 times the female rate (4.5:1) of 6.5/ 100 000 population (95% CI: 4.7–8.2) in older adolescents (15–17 year old). Adolescent males were significantly more likely (61.2%) to die in a public space compared to female adolescents (39.3%) but more adolescent females (48.4%) died at home compared to adolescent males (32.9%). Adolescent females (42.1%) were mainly killed by a family member or intimate partner while adolescent males were predominantly (58%) killed by an acquaintance.

**Conclusion:**

We found a distinct gender pattern for adolescent homicide in South Africa. This pattern appears to be driven by gender norms that support violence. South Africa requires an investment in developing evidence informed interventions to reduce violence.

## Introduction

Yearly, large numbers of young people, mainly adolescent males, die due to interpersonal violence. In 2015, it is estimated that 51,000 adolescents died due to homicide globally, a number that far exceeds the number of deaths of adolescents from armed conflict (30,000) which included war and civil unrest [1]. The global adolescent homicide rate for 2015 is estimated at 4.3 per 100 000 population among adolescents aged 10–19 years, with a distinct gendered dimension which mirrors the adult homicide pattern [1]. The homicide rate for adolescent males (6.8 per 100 000) aged 10–19 years, is more than four times the rate for adolescent females with some regional variation [1]. The region with the highest adolescent homicide rate is reportedly Latin America and the Caribbean with an estimate rate of 22.1/100 000 per population, followed by West and Central Africa (5.4 per 100 000 population), then East and Southern Africa (4.1 per 100 000 population)[1].

Data from South Africa shows that homicide contributes substantially to the mortality burden with similarly higher rates among young men in countries with high levels of violence [2]. A dedicated injury mortality study reflecting 2009 data reported an estimated overall adult homicide rate of 38.4 per 100 000 population with a significantly higher rate of 56.7 in the 15–29-year age group [2]. This study also showed the gender pattern of homicide with a male-to-female ratio of six male deaths for every female death (67.4/100 000 vs 11.3/100 000). Although the age groups are not directly comparable, the South African study suggests a more pronounced gender divide compared to global estimates, but similar to the Americas [3]. South Africa has seen a substantial decrease (2000 to 2009) in the homicide rate for both men and women since the introduction of gun control legislation (Firearms Control Act No 60 of 2000) in 2000, which makes provision for more responsible gun ownership through stricter eligibility and competency requirements [2]. Nevertheless, the current homicide rates are still excessive, and place South Africa among the most violent countries globally [2,4].

Interpersonal violence particularly among young men has been recognised as a public health concern, globally. The United Nations General Assembly adopted the Sustainable Development Goals (SDGs) in 2015, and for the first time they have a specific focus on addressing interpersonal violence as a challenge to the overall development of a nation [5]. This is part of Goal 16 (Peace and Justice), which also acknowledges the cost of not taking action [5]. This goal includes a target to reduce all forms of violence and torture against children and recognises the need to prevent violence early in the life course. Although the conceptualisation of interpersonal violence among youth incorporates ages 10–29 years [6], this masks the violence experienced in the adolescent age group. There is an increasing risk for victimisation during adolescence but this only becomes evident with an adolescence focussed analysis [1]. Developing an understanding of the pattern of homicide victimisation and perpetration in this neglected age group is important and has the potential to stem the tide of interpersonal violence.

Few studies in South Africa have described the national pattern of adolescent homicides. Homicides during this age group are often classified as youth violence and combined with older young adults [6,7] and masks important factors inherent in this age group that potentially drive the pattern of violence and homicide. The pattern of youth violence clearly highlights the risk of young men becoming both victims and perpetrators of violence, with devasting consequences such as death and long-term disability. The victim-perpetrator relationship is of importance as violence among peers and dating violence are prevalent during adolescence [1]. Although young women are less likely to be victims of homicide in this age group, they nevertheless experience violence of a gendered nature in dating relationships, through peer relationships and sexual violence [7]. Violence against children in South Africa is considered endemic with 34.4% of children reporting experiences of physical abuse and 19.8% sexual abuse [8]. Children’s risk of violence also drives the pattern of child homicide with South Africa showing a distinct pattern—child abuse and neglect deaths mainly occur in children under the age of 5 years; while rape homicides occur mainly in girls across their life course [9].

The 2009 National Child Homicide study [9] provides an opportunity to describe the homicide patterns of South African adolescents. In this paper, we describe the prevalence of and gender difference in adolescent homicide in South Africa in 2009. We also explore the social and demographic characteristics of the victims and perpetrators. We used the United Nations Convention on the Rights of the Child (UNCRC) definition of a child as under the age of 18 years and define adolescence as the age group 10–17 years. Based on the literature review, we acknowledge that adolescence is often defined as 10–19 years, but the study collected data until the age of 17 years in keeping with the UNCRC definition of the “child”. Understanding how violence differentially affects adolescent girls and boys is important as it will provide us with better insight into possible opportunities to work towards preventing violence and work towards achieving the SDGs.

## Methods

We conducted a retrospective national mortuary-based prevalence study of all child homicides and combined data from mortuary records, police and police interviews. Although South Africa has a relatively robust vital registration system, challenges have been noted with cause-of-death data; namely a high proportion of ill-defined deaths and an inaccurate injury-death profile [10]. Previous studies have shown the utility of conducting reviews of autopsies at state medical legal laboratories to improve the accuracy of homicide rates in South Africa [10,11]. We therefore identified child homicide cases at state laboratories as all unnatural deaths in South Africa must undergo a post-mortem examination at a state laboratory according to the South African Inquest Act of 1959 [12]. We included all cases that presented at laboratories between 1 January 2009 and 31 December 2009. All laboratories operational in 2009 were stratified based on the number of post-mortems performed for 2009 and formed during the sampling frame. There were three strata: small < 500, medium 500–1499 and large >1499, with 20 laboratories randomly selected in strata one, 13 in strata two and five in strata three (see Table 1). Thirty-eight laboratories were selected proportionally from the 123 laboratories operating for 2009, the sample therefore ensuring precision for national estimates. For this analysis we focus on a sub-sample of children, the adolescent group aged 10–17 years.

**Table 1:** The Sampling Fraction based on the operating medical legal laboratories in South Africa in 2009.

Number of Autopsies per Annum	Number of Medical Legal Laboratories (N)	Sample (N)	Sampling Fraction
>1 499	9	5	55.5
500–1 499	33	13	39.4
<500	81	20	24.7
Total	123	38	30.9

Data Source: National Department of Health, Forensic Pathology Services 2010

Ethical approval for the study was granted by the Ethics Committee of the South African Medical Research Council (EC09-021). This study reviewed patient records of deceased children and the ethics committee waivered the need to obtain parental consent in order to minimise further emotional harm. Further approval and access to mortuary data were obtained from the National Department of Health and permission to access police data were obtained from the South African Police Service. Patient records were reviewed at mortuaries without identifying details to maintain confidentiality and anonymity. We gathered police record numbers at mortuaries to be able to link cases for logistical purposes. Police record numbers were only used for this purpose and all record numbers were removed before data analysis.

For this analysis we focus on a sub-sample of children, the adolescent group, aged 10–17 years, in line with the definition of a child as defined by Article 1 of the UNCRC, as a person under 18 years of age. Child homicide was defined as the death of a child for which another person was responsible, based on the outcome of a police investigation and as stipulated by the Inquest Act of 1959 [12]. We used a phased approach to data collection: firstly, cases were identified through death registers at the sampled MLLs; secondly, post-mortem reports and mortuary records were used to verify cause of death as homicide. Finally, police interviews were conducted for further verification of homicide. We excluded cases where the police interview indicated accidental or unintentional death. We considered fatal child abuse and neglect, as well as rape homicide as a subset of child homicide, this is further described elsewhere [9]. For this paper we combine all forms of child homicide and only report on child homicide as one category.

Phase one was the identification of child homicide via mortuary death registers. The mortuary files of identified cases were drawn by mortuary staff for review by a researcher. Data were collected using a standardised pre-tested data collection form to extract data from the post-mortem reports. The data collected included the victim’s demographic characteristics (including age, gender, location of the crime, rural/urban), cause of death (gunshot, stab, blunt force, strangulation, smothered, multiple injuries), location of injury, pathology (pregnancy and sexual assault), toxicology data (blood alcohol and poisoning) and the police case number and police station. Police data were collected through the use of the police case number and identified police station. Telephonic or face-to-face interviews were conducted with the investigating officer to ascertain circumstances around the death, victim-perpetrator (or suspect) relationship, information about the perpetrator or main suspect (age, gender, employment, history of previous convictions), criminal outcome of the case and whether there was a suspicion of child abuse. All cases where there was evidence of sexual assault as part of the homicide were included as part of the definition of fatal child abuse and we included cases where it was confirmed by a police investigation. The police investigation data were crucial to provide data on the profile of the perpetrator, victim-perpetrator relationship and outcomes of the criminal case as there are no other data sources to gather this information from in South Africa. The data were analysed to explore gender differences in victimisation patterns amongst adolescents as well as to develop a profile of perpetrators and the outcomes of cases according to the gender of victims. We used the age cut-off of 20 years for perpetrators to match the UNICEF definition of adolescents (10–19 years) [1].

Data were analysed using Stata version 13 and the survey design and sampling weights of mortuaries were considered in the analysis. We calculated adolescent homicide rates for female and male children 10–14 years and 15–17 years and overall rates for 10–17 years, using adjusted population data released by Statistics South Africa (2017) for the 2001 census population estimates as the denominator and the specific age estimates from the study as the numerator. Descriptive statistics (means and proportions) were calculated, with adjustment for stratification, and weighting of mortuaries based on the differential allocation of sample weights by strata. Categorical variables were compared using Pearson’s chi-squared tests, using the command for survey data. Standard errors and 95% confidence intervals were calculated using methods for complex sample surveys (Taylor linearization) [13].

## Results

We identified 674 (95% CI: 520–823) adolescent homicides for 2009, with 520 male and 154 female homicides. The overall adolescent homicide rate per 100 000 population 10–17 years was 8.2 (95% CI: 6.3, 10.1), see Table 2. We found a higher adolescent homicide rate among males, 12.6 (95% CI: 9.5–15.6) compared to females, 3.8 (95% CI: 2.7–4.8) per 100 000 population 10–17 years. This gender disparity increased in the older adolescent age group (15–17 years), with the male homicide rate 27.9/100 000 population (95% CI: 20.3–35.5), nearly five times the female rate (4.5:1) of 6.5/100 000 population (95% CI: 4.7–8.2).

**Table 2:** Estimated age-specific homicide rates for adolescents 10–17 years (weighted estimates).

Age Group	Number of Homicides		Pop Data	Rate per 100000 (95% CI)		Male		Pop Data	Rate (95% CI)		Female		Pop Data	Rate (95% CI)	
	%	95% CI		Rate	95% CI	%	95% CI		Rate	95% CI	%	95% CI		Rate	95% CI
**10–14 yrs**	146	(116–175)	5184161	2.82	(2.24–3.38)	88	(64–112)	2598391	3.39	(2.46–4.31)	58	(35–80)	2585771	2.24	(1.35–3.09)
**15–17 yrs**	529	(392–665)	3030172	17.46	(12.93–21.95)	432	(315–550)	1548418	27.90	(20.34–35.52)	96	(70–121)	1481754	6.48	(4.72–8.17)
**Total Adolescent (10–17 yrs)**	675	(520–828)	8214333	8.22	(6.33–10.08)	520	(393–648)	4146809	12.54	(9.48–15.62)	154	(111–197)	4067525	3.79	(2.73–4.84)

Table 3 presents the socio-demographic and incident data for adolescent homicides. The mean age shows that adolescent male homicide victims were slightly older (15.4 years) than females (14.7 years). We found that adolescent males were more likely to die in urban areas compared to adolescent female homicide victims. Adolescent males (61.2%) were significantly more likely to die in a public space compared to 39.3% of adolescent females. Adolescent females were more (48.4%) likely to die at home compared to 32.9% of adolescent males. More than a third of adolescent females (38.1%) were raped and killed, while a rape homicide was not common among young males (1.3%). Strangulation deaths were mainly a feature of homicide among adolescent females (11%) rather than adolescent males (1.1%). Nearly half (47.1%) of adolescent males were stabbed compared to 23.5% of adolescent females, while gunshot deaths were similar among male (20.8%) and female (23.9%) adolescent homicides. Blood alcohol specimens were taken in just over half of the adolescent victims (51.4% males vs. 52.6% females) with no gender difference. Blood alcohol results were not available for 38 of the victims. Where blood alcohol results were available, a similar proportion (39.8%) of adolescent male victims had a positive blood alcohol result compared to (36.9%) adolescent female victims. For those with a positive blood alcohol result, the mean blood alcohol content (BAC) among male adolescent victims, was nearly double (0.10g/100ml) the BAC of female victims (0.06 g/100ml), but the mean BAC difference was not significant.

**Table 3:** Frequencies of socio-demographic and pathology outcomes for adolescent homicide victims by sex (weighted estimates).

	Adolescent Homicides N = 674		Male n = 520		Female n = 154		p-value
**Victim Age**	%/mean	95% CI	%/mean	95% CI	%/mean	95% CI	
Mean Age	15.4		15.6		14.7		
Standard Deviation	1.67		1.51		2.05		
Median Age	16		16		15		
**Location of Crime**	** **		** **		** **		
Urban Area	52.3	(39.2–64.9)	54.4	(39.3–68.9)	45.0	(36.0–54.4)	0.133
Small Town/Rural	47.7	(35.0–60.8)	45.6	(31.1–60.9)	55.0	(45.6–64.0)	
**Crime Scene**							
Home/Other home	36.5	(31.3–41.9)	32.9	(26.9–39.5)	48.4	(39.2–57.7)	0.004
Public Space	56.1	(51.3–60.8)	61.2	(54.7–67.3)	39.3	(29.4–50.1)	
Other Place	5.6	(4.2–7.5)	4.9	(3.3–7.2)	8.0	(4.1–15.0)	
Unknown	1.8	(0.9–3.5)	1.0	(0.3–3.1)	4.3	(1.9–9.7)	
**Sexual Homicide**							
Yes	9.4	(6.2–14.0)	1.3	(0.5–3.3)	38.1	(28.7–48.6)	< 0.000
No	90.6	(85.9–93.8)	98.7	(96.7–99.5)	61.9	(51.4–71.3)	
**Mean Blood Alcohol Content (g/100ml)** **for the Sample with a +ve Blood Alcohol**	0.09	(0.06–0.12)	0.10	(0.06–0.13)	0.06	(0.01–0.10)	0.2891
**Cause of Death**							
Gunshot	21.5	(18.3–25.2)	20.8	(17.9–24.1)	23.9	(17.8–31.2)	< 0.000
Stab	41.7	(34.9–48.8)	47.1	(39.9–54.4)	23.5	(16.8–31.2)	
Blunt Force	17.0	(13.3–21.54)	16.6	(11.7–23.2)	18.4	(12.3–26.6)	
Strangled	3.4	(1.7–6.6)	1.1	(0.4–2.9)	11.0	(5.6–20.6)	
Smothered	0.4	(0.1–1.8)	0		1.6	(0.3–7.9)	
Multiple Injuries	3.8	(2.2–6.5)	4.5	(2.6–7.6)	1.6	(0.3–7.6)	
Other	8.6	(5.6–12.9)	7.2	(4.3–11.7)	13.3	(7.9–21.5)	
Undetermined	3.6	(2.3–5.6)	2.7	(1.5–4.9)	6.6	(3.3–12.8)	

Table 4 presents the perpetrator characteristics and conviction outcomes. Overall, just over two thirds (68.6%) of perpetrators of adolescent homicide were identified with a lower proportion among female victims (58.6%) compared to male victims (71.5%). The mean age (25.4 years) of perpetrators showed that most were young adults, but perpetrators of adolescent male homicides were significantly younger (22.2 years) than perpetrators of adolescent female homicides (26.8 years) (*p* = 0.0161). In addition, significantly more adolescent male homicide perpetrators (50.9%) were under the age of 20 years than perpetrators of adolescent female homicide (18.1%).

**Table 4:** Frequencies of perpetrator characteristics for adolescent homicides (weighted estimates).

	Adolescent Homicides N = 674		Male n = 520		Female n = 154		p-value
**Perpetrator was Identified**	%/mean	95% CI	%/mean	95% CI	%/mean	95% CI	
Identified	68.6	(62.6–73.9)	71.5	(65.2–77.1)	58.6	(50.3–66.4)	0.0003
Suspected Only	7.7	(5.6–10.7)	5.2	(3.4–8.0)	16.3	(10.3–24.8)	
Remained Unidentified	23.7	(19.0–29.1)	23.3	(18.7–28.6)	25.1	(17.7–34.3)	
**Mean Perpetrator Age**	25.4		22.2		26.8		
**Perpetrator Under 20 Years Old**							
**Yes**	43.7	(37.6–49.9)	50.9	(45.4–56.5)	18.1	(10.9–28.4)	< 0.000
**No**	56.3	(50.1–62.3)	49.1	(43.6–54.5)	81.9	(71.6–89.1)	
**Perpetrator Relationship with Victim**							
Family Member	13.1	(11.1–15.5)	11.2	(9.1–13.8)	19.5	(15.3–24.6)	< 0.000
Acquaintance	51.5	(45.6–57.4)	58.0	(52.4–63.3)	30.2	(22.8–38.9)	
Intimate Partner	6.4	(4.1–9.7)	1.5	(0.7–3.4)	22.6	(15.2–32.1)	
Stranger	6.5	(4.9–8.5)	7.3	(5.2–10.2)	3.9	(1.7–8.6)	
Other	17.7	(13.3–23.2)	19.0	(13.8–25.5)	13.6	(9.4–19.1)	
Unknown	4.8	(3.3–6.9)	3.1	(2.0–4.9)	10.3	(5.6–18.2)	
**Multiple Perpetrators**							
Yes	25.5	(20.5–31.3)	30.0	(24.5–36.1)	11.0	(5.1–22.2)	0.0049
No	56.9	(52.3–61.4)	53.6	(48.2–58.9)	67.8	(58.0–6.2)	
Unknown	17.55	(12.6–23.9)	16.4	(11.7–22.6)	21.2	(13.9–30.9)	
**Convicted**							
Yes	21.2	(17.6–25.2)	18.9	(14.6–24.3)	28.5	(23.8–33.8)	0.02
Awaiting Trail	21.2	(17.5–25.5)	22.2	(18.2–26.9)	17.8	(12.6–24.4)	
No	57.6	(53.9–61.2)	58.8	(54.5–62.9)	53.7	(47.2–60.2)	

The relationship between victim and perpetrator differ substantially by gender. Most adolescent women (42.1%) were killed by a family member or intimate partner while only 12.2% of adolescent males were killed by them. Perpetrators were mainly known to the adolescent, with more adolescent males (58%) compared to females (30.2%) killed by an acquaintance. Multiple perpetrators were significantly more common among adolescent male homicides (30.0%) than adolescent female homicides (11.0%). Overall, the conviction rate was 21.2% and was lower for adolescent male homicides (18.9%) compared to adolescent female homicides (28.5%).

## Discussion

Our study showed that South Africa had nearly 700 adolescent homicides for 2009, with an adolescent homicide rate (8.2/100 000 population) almost double the global adolescent homicide rate of 4.3/100 000 [1]. Of note, the global rate includes the age group 10–19 years, while our data only included adolescent homicides from 10–17 years making direct comparison difficult. Importantly, we found a distinct age and gender difference in the pattern of adolescent homicide. A marked increase in the male homicide rate is observed as age increased, a rate of 3.39/100 000 males 10–14 years compared to 27.9/100 000 males 15–17 years old. However, for women the age differences were less pronounced, increasing from 2.24/100 000 females 10–14 years to 6.48/100 000 females 15–17 years. This pattern is similar to findings from Brazil where it was also shown that the increase in male adolescent homicide across age groups far exceeded that of females [14]. Furthermore, the increased homicide rates for males in late adolescence continues and peaks in early adulthood (20–29 years) with a decline thereafter [14].

Few studies describe the gender pattern of adolescent homicide as young men bear the burden of homicide globally. Our study showed that although male homicides are overrepresented in adolescence it assumes a distinct gender pattern. Adolescent males were killed in public spaces, by an acquaintance, killed by a stab wound and had higher mean blood alcohol content than female adolescent victims. This pattern suggests that adolescent male deaths are possibly related to engagement in anti-social behaviour with some evidence pointing to exposure and experience of violence during childhood as contributing factors [15]. In addition, data from elsewhere shows that persistent structural inequalities continue to limit young men’s opportunities and can lead to them participating in crime and violence [16]. In certain contexts, it has been found that gang involvement can provide young men with the recognition and respect they do not receive elsewhere [17]. The dominant constructions of masculinity in South Africa has been shown to emphasise competition between men, ready use of violence to defend honour and heavy use of alcohol which often fuels violent behaviour [18]. It is critical to develop an understanding of what works to prevent violent behaviour among young men in communities. Although the evidence base is scant, gun control and alcohol restrictions are among the factors that have been shown to reduce homicides [19]. There are more than likely other factors that have not been identified that also play a role in reducing homicide.

Adolescent female homicides were more likely to occur in the home, with the perpetrator being someone close to the female adolescent. This appears to be different to the dynamics in adolescent male homicides that point to males often being participants in the violence that results in death, for instance engaging in fights that become lethal. Our study shows that young women are predominantly victims rather than participants in violent acts. In fact, nearly a quarter of these adolescent girls were killed in the home by an intimate partner and another 20% were killed by a family member who were mainly male, suggesting a gender-based violence pattern driving the homicide of adolescent women. Intimate femicide is the leading cause of female homicide in South Africa and considered to be the most severe form of gender-based violence [20]. In addition, a large number of these adolescent female homicides were sexual homicides, which are common in South Africa [21]. Gender-based violence appears to be an important common underlying feature in these female adolescent homicides and core to our understanding thereof. There are common risk factors that underscore perpetration of intimate partner violence which include: gender and relationship factors as well as experiences of childhood trauma; alcohol misuse and depression; low education; poverty and involvement in gangs and fights with weapons [22]. Furthermore, gender inequality and gendered social norms that promote men’s dominance and control over women and allow for the tolerance of violence against women and young girls [18]. Our study shows that it is mainly adult men who kill these young women. Research with men who killed an intimate partner has shown how early traumatic experiences influence these men’s psychological vulnerabilities combined with social context which allows for the occurrence of such murders [23]. Family level psycho-social programmes focussing on strengthening families to provide nurturing care and improving parenting skills are critical to prevent early traumatic experiences that contribute to perpetration of violence by men [24].

Our study has limitations: we collected data on homicides that occurred in 2009, while it appears to be old, data were only collected once cases had been through the legal system. In addition, this is the only data set linking mortuary data and police data on child homicides (used the adolescent data for this analysis) from mortuaries and linked cases with police data to obtain perpetrator information and conviction outcomes and provides a unique level of detail. Perpetrators remain unidentified in 25.1% of the cases. Perpetrator information allowed us to determine the context of the incident, for example whether the crime was due to male-on-male interpersonal violence, a family member or caregiver (child abuse) and in the case of females, an intimate partner. Due to the hidden nature of intimate partner violence, it may be that our study underestimates the intimate partner homicide among adolescent girls. Furthermore, bloods were taken to test for blood alcohol content in just over half of adolescents, but we had missing results in 37% of the cases where blood alcohol was taken. However, we do not expect this to be a biased sample of missing specimens, thus the overall findings are probably fairly valid.

Young men bear the brunt of adolescent homicides in South Africa which is no different to other countries with high levels of violence [1]. The adolescent homicide pattern mirrors that of adults, with a distinct gendered pattern and adolescent male homicides exceeding that of adolescent females. We argue that the homicide of both young men and women are driven in different ways by social norms that support the use of violence both within the home and in the community [19]. South Africa has joined the Global Partnership to End Violence Against Children as a “pathfinder” country at the end of 2017. It is imperative that South Africa develop an understanding of what works to prevent violent behaviour among young men to reduce male violence in communities and men as perpetrators of violence in intimate relationships. South Africa should also invest in developing evidence-based strategies to reduce violence in families and reduce violence against children to lower the risks for perpetrating violence in early childhood. These are critical steps to attain the SDG goal to work towards the creation of safer communities for all.
